# Suppression of the Ubiquitin Pathway by Small Molecule Binding to Ubiquitin Enhances Doxorubicin Sensitivity of the Cancer Cells

**DOI:** 10.3390/molecules24061073

**Published:** 2019-03-19

**Authors:** Thanh Nguyen, Minh Ho, Kyungmin Kim, Sun-Il Yun, Pushpak Mizar, James W. Easton, Seung Seo Lee, Kyeong Kyu Kim

**Affiliations:** 1 Department of Molecular Cell Biology, Sungkyunkwan University School of Medicine, Suwon 440-746, Korea; ntth@skku.edu (T.N.); honguyenanhminh@gmail.com (M.H.); sipoppy@hanmail.net (S.-I.Y.); 2 Genome Integrity and Structural Biology Laboratory, NIEHS, National Institutes of Health, Research Triangle Park, NC 27709, USA; sbl.kmkim@gmail.com; 3 Chemistry, Faculty of Engineering & Physical Sciences, University of Southampton, Highfield, Southampton SO17 1BJ, UK; P.Mizar@soton.ac.uk (P.M.); jwe1g13@soton.ac.uk (J.W.E.)

**Keywords:** ubiquitin, protein-protein interaction, inhibitor, deubiquitinase, ubiquitination

## Abstract

Development of inhibitors for ubiquitin pathway has been suggested as a promising strategy to treat several types of cancers, which has been showcased by recent success of a series of novel anticancer drugs based on inhibition of ubiquitin pathways. Although the druggability of enzymes in ubiquitin pathways has been demonstrated, ubiquitin itself, the main agent of the pathway, has not been targeted. Whereas conventional enzyme inhibitors are used to silence the ubiquitination or reverse it, they cannot disrupt the binding activity of ubiquitin. Herein, we report that the scaffolds of sulfonated aryl diazo compounds, particularly Congo red, could disrupt the binding activity of ubiquitin, resulting in the activity equivalent to inhibition of ubiquitination. NMR mapping assay demonstrated that the chemical directly binds to the recognition site for ubiquitin processing enzymes on the surface of ubiquitin, and thereby blocks the binding of ubiquitin to its cognate receptors. As a proof of concept for the druggability of the ubiquitin molecule, we demonstrated that Congo red acted as an intracellular inhibitor of ubiquitin recognition and binding, which led to inhibition of ubiquitination, and thereby, could be used as a sensitizer for conventional anticancer drugs, doxorubicin.

## 1. Introduction

Ubiquitination is one of the most abundant post-translational modifications (PTM) [1,2], which plays a crucial role in cell homeostasis. The functional diversity of the highly conserved 76 amino acid long polypeptide, ubiquitin, lies within the large variety of poly-ubiquitin chains that signal either degradative or non-degradative pathways [3]. After activation by the activating enzyme (E1), the conjugating enzyme (E2) attaches the C-terminal glycine of the first ubiquitin polypeptide to a lysine residue of another ubiquitin polypeptide, via an isopeptide bond. This process may be repeated until the growing poly-ubiquitin chain reaches an appropriate length, before finally being attached to a target substrate by the ligase enzyme (E3) [2]. With linkages through seven different lysine residues (K6, K11, K27, K29, K33, K48, K63) and a head-to-tail linkage, there are a total of eight different types of poly-ubiquitin linkages with different topologies [4,5,6]. Furthermore, poly-ubiquitination may occur in either a homogenous or a mixed fashion, which means there may be more than one linkage types, and there can be branched chains. These combinations of many different types of linkages result in the diversity of topologies, which are likely to be specific for particular functions [7,8]. This PTM is also reversible depending on the cellular requirements, adding another layer of complexity in ubiquitin pathways. This reverse reaction of ubiquitination is carried out by a deubiquitinase (DUB) enzyme that hydrolyzes the poly-ubiquitin chain into monomers or detaches it from the target protein [9,10].

Among the different types of poly-ubiquitin topologies, K48 is the most characterized poly-ubiquitin chain. It has been well documented that K48 poly-ubiquitination marks a protein for degradation by the proteasome [5]. Besides K48, K6 and K11 have also been implicated in proteasome degradation, whereas K63 and linear ubiquitination are involved in DNA damage repair and NF-κB signaling pathways [3,4,11]. Mono ubiquitination was suggested to control the membrane trafficking, endocytosis, and transcriptional regulation [12,13]. In contrast to the well-known function of the homogenous poly-ubiquitin chains, very little is known about the function and regulated pathways of mixed and branched ubiquitin chains. Although ubiquitin signaling is usually understood as an ATP dependent pathway involving multiple-enzymes, the function of ubiquitin should be viewed not only from ubiquitination but also from the aspect of recognition of ubiquitin by cognate receptors through ubiquitin recognition motifs or domains, i.e., binding activity of ubiquitin [14,15]. Accordingly, ubiquitin binding has been proven important in coupling ubiquitination with the control of protein–protein interaction, nuclear localization, degradation, and immune response [14,15,16], including extracellular signaling, such as the CXC chemokine receptor 4 (CXCR4) pathway to control apoptosis, immune responses or cancer progression [17,18,19].

The malfunction of the ubiquitin pathway has been implicated in several diseases including neurodegenerative disorder [20] and cancers [21,22]. Particularly, the dysregulation of the ubiquitin pathway was found to lead to either reduced drug efficacy [23] or development of drug resistance [24] in several cancers under chemotherapy. It has been well documented that in both treatment by doxorubicin (Dox), a genotoxic anticancer drug, and in Dox-resistant cancer cells, the ubiquitination system is activated [25,26,27] leading to either enhancement of the activation of NF-κB by promoting degradation of the inhibitor IκBα [28] or activation of the DNA damage repair system [29] resulting in reduced Dox sensitivity. The activation of the ubiquitination system was also reported in radiotherapy of several cancers, leading to fast recovery of the tumors post-radiotherapy [30]. Therefore, studying the development of small molecule inhibitors that target this pathway is considered a novel promising strategy to treat the diseases or to enhance conventional anticancer therapeutics [21,30]. In general, every step in the cycle can be targeted for development of such drugs [31]. Thus far, E1, E2, E3, DUBs, and proteasome have been targeted and several inhibitors developed [31,32]. Among them, the proteasome inhibitor Bortezomib, a commercial anticancer drug approved by the US FDA can work either alone or in combination with conventional anticancer drugs [33,34] to enhance the efficacy depending upon tumor types.

In 2016, we described a new inhibitor in this pathway, which bound to the ubiquitin polypeptide [35]. Chicago Sky Blue 6B (CSB6B), an azo dye compound, was identified as an inhibitor to a deubiquitinase in a high throughput screening, but later revealed not to bind to deubiquitinase, but to ubiquitin. It was subsequently shown that CSB6B shut down all the pathways involving ubiquitin, including ubiquitination, deubiquitination and ligand-receptor interaction [35]. As CSB6B is a highly sulfated molecule, it could not penetrate the cell membrane, but instead, successfully inhibited the activity of extracellular ubiquitin. As a representative example, CSB6B inhibited the action of ubiquitin in the CXCR4 pathway. Based on this, we have further explored the structure-activity relationship of the molecular scaffold of CSB6B, and herein, we report another inhibitor that acts similarly, but intracellularly. We present in this report that Congo red (CR) could penetrate the cell membrane, allowing it to disrupt intracellular protein-protein interactions (PPI) between ubiquitin and ubiquitin processing enzymes and hence diminish the activities of the ubiquitin pathways inside the cell. We also propose a binding mode of ubiquitin with CR from the NMR mapping experiments and demonstrate the impact for this class of inhibitor through its antagonizing effect on Dox-mediated activation of the ubiquitination system to increase Dox sensitivity.

## 2. Results

### 2.1. Sulfonated Aryl Diazo Compounds Inhibit DUBs by Binding to Ubiquitin

**Figure 1:**
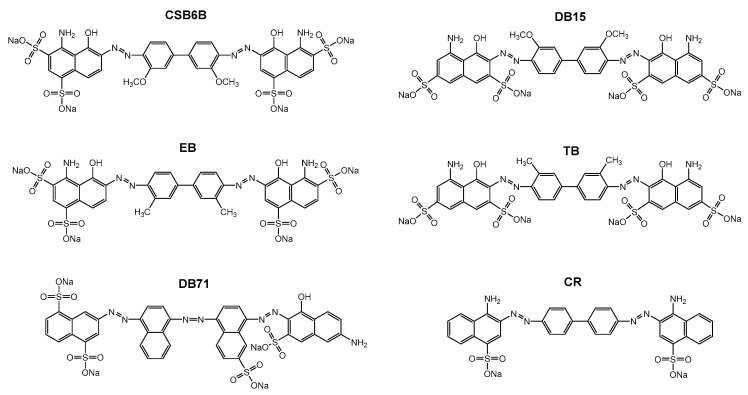
Structure of CSB6B and five sulfonated aryl diazo compounds that have structures similar to that of CSB6B, Evans blue (EB), Direct blue 15 (DB15), Trypan blue (TB), Direct blue 71 (DB71), and Congo red (CR).

**Figure 2:**
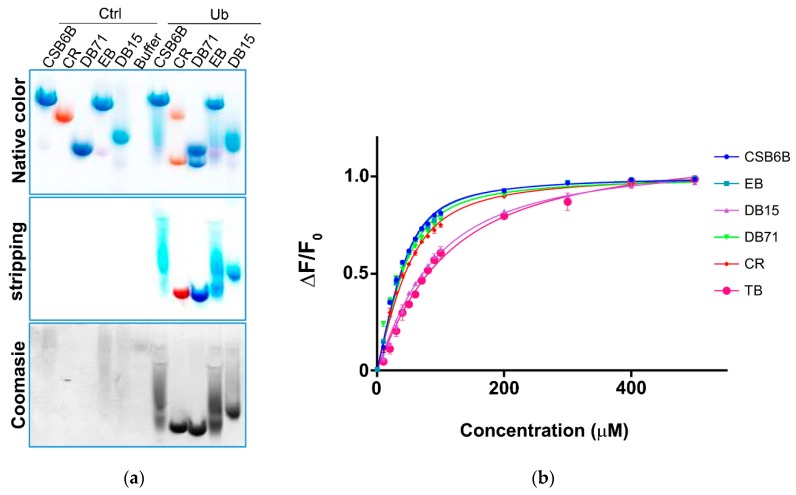
Binding of sulfonated aryl diazo compounds to ubiquitin. (**a**) Visualization of compounds alone (Ctrl) and bound to ubiquitin (Ub) in native-PAGE before (top, native color) and after (middle, stripping) stripping with de-staining solution. Protein bands were stained by Coomassie blue (bottom). (**b**) Intrinsic fluorescence anisotropy of ubiquitin upon treatment with various CSB6B analogs.

After the discovery of Chicago sky blue 6B (CSB6B) as an inhibitor of ubiquitin, we tested and observed the inhibitory activities of various structurally similar sulfonated aryl diazo compounds using the same assay method as described previously [35]. Aryl diazo compounds included Evans blue (EB), Congo red (CR), Direct blue 15 (DB15), Direct blue 71 (DB71), and Trypan blue (TB), in addition to CSB6B (Figure 1). It was observed that these compounds inhibited USP5 by binding to ubiquitin instead of binding to the enzyme as previously [35]. On the native polyacrylamide gel electrophoresis (N-PAGE), the mobility shifts of both ubiquitin and the compounds in the mixtures in comparison with free compounds or ubiquitin were clearly observed (Figure 2a).

### 2.2. Structure-Activity Relationship of Sulfonated Aryl Diazo Compounds

**Figure 3:**
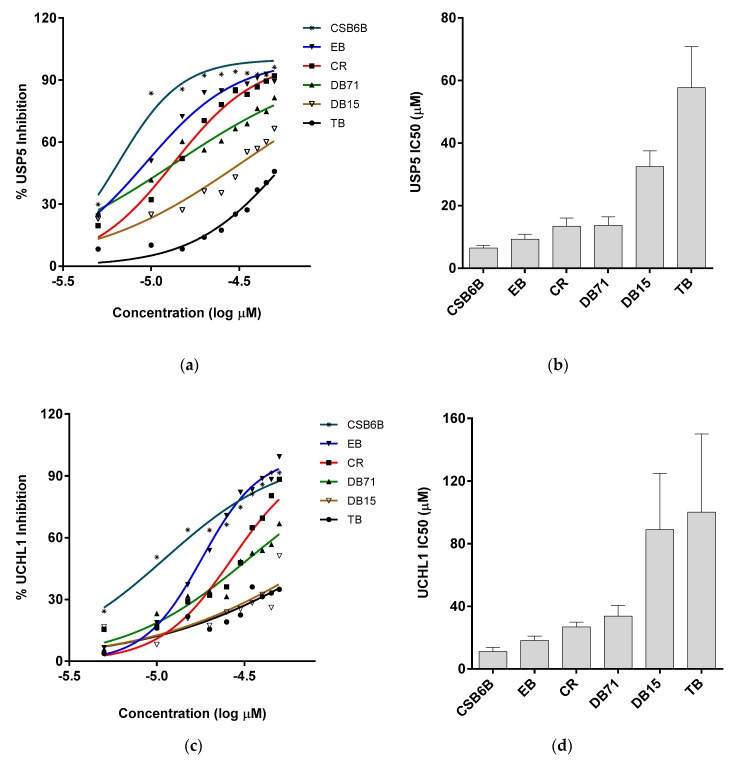
Inhibitory activities of ubiquitin-binding compounds on deubiquitinases’ (DUBs) activities. Activities of USP5 (**a**,**b**) and UCHL1 (**c**,**d**) upon treatment with different compounds. (**a**,**c**) Inhibitory activity of ubiquitin-binding compounds at different concentration are plotted as percent inhibition using the quantified band intensities. (**b**,**d**) IC_50_ values of different compounds against USP5 and UCHL1 analyzed from Figure 3a,c are plotted, respectively.

In order to establish structure–activity relationships within this class of compounds, dissociation constants (K_d_) for the binding affinity between each compound and ubiquitin, and half maximal inhibitory concentration (IC_50_) values against two DUBs, USP5 specific for unanchored polyubiquitin chains [36] and ubiquitin c-terminal hydrolase L1 (UCHL-1) specific for processing of ubiquitin precursors, [37] were measured. Thus, in the intrinsic fluorescence quenching assay, EB, CR, and DB71 showed binding affinities similar to that of CSB6B, whereas DB15 and TB showed substantially lower binding affinities (Figure 2b and Table 1). Consistent with this result, EB, CR, and DB71 showed considerably lower IC_50_ values against the activity of both USP5 (Figure S1a, Figure 3a,b and Table 2) and UCHL1 (Figure S1b, Figure 3c,d and Table 2) on the GST-Ubiquitin-HA, compared to that of DB15 and TB.

**Figure 4:**
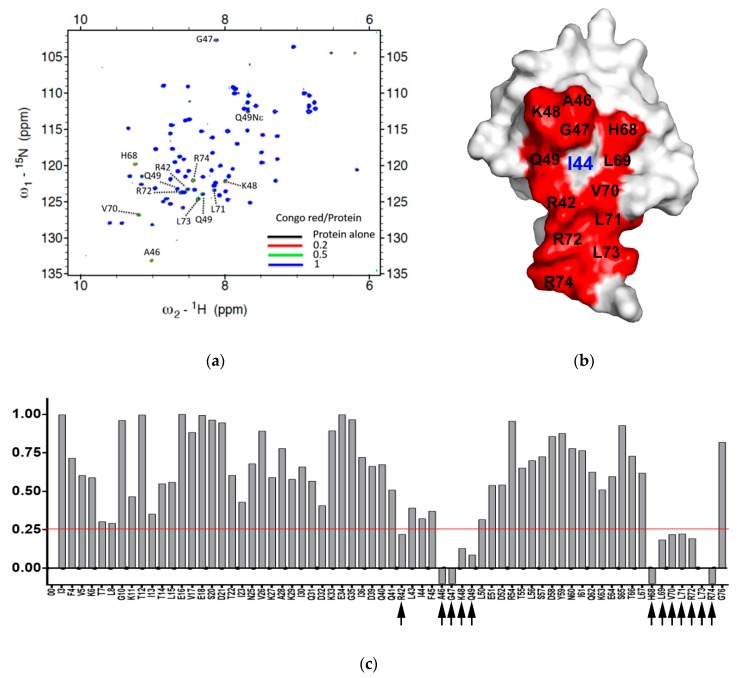
The interacting surface of ubiquitin used for binding with Congo red. (**a**) ^1^H,^15^N-HSQC spectra of ^15^N-labeled ubiquitin upon treatment of Congo red at different concentration: Black, 100 μM ^15^N-ubiquitin alone, red, with 20 μM Congo red, green, with 50 μM Congo red, blue, with 100 μM Congo red. The peaks with chemical shift or line broadening are indicated by dotted lines and labeled. (**b**) The ubiquitin-interacting surface used for CR binding. The residues disturbed upon treatment with CR were indicated in red color with labels on the surface-filling model of Ub. The center of the canonical hydrophobic patched, Ile44 is labeled in blue. (**c**) Normalized intensity reduction ratio (I_N_) of each residue upon treatment of Congo red (CR) at 1:1 ratio was compared. The value below zero (−0.1) indicates the disappearing residues. Black arrow indicates the residue whose intensity remains less than 30% (I_N_ < 0.25, red line).

**Figure 5:**
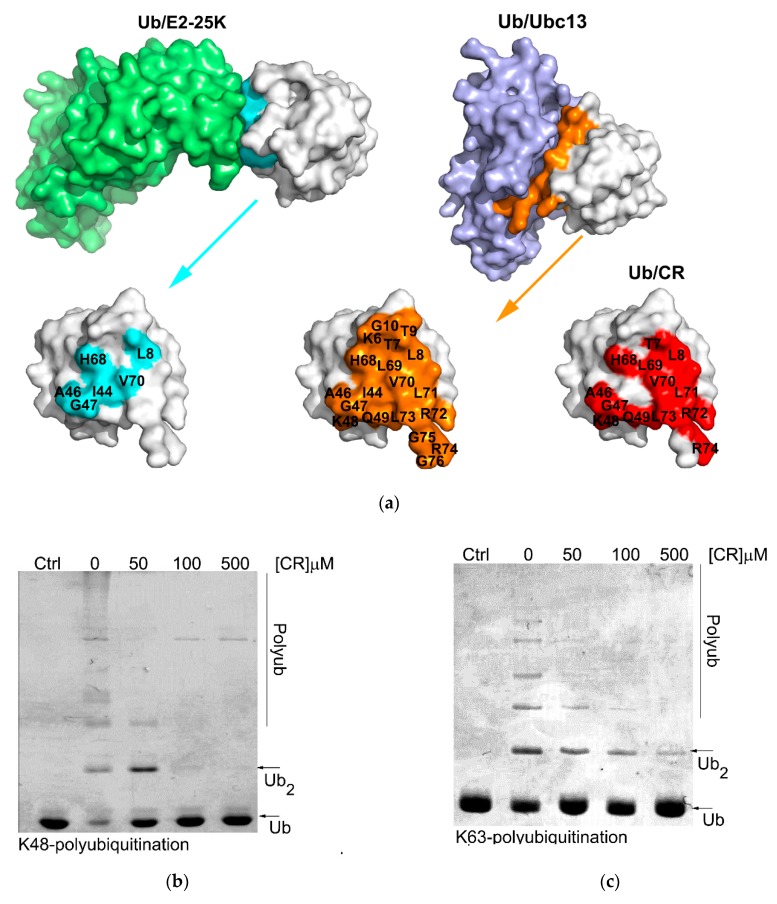
CR inhibits K48- and K63- polyubiquitination in vitro. (**a**) Interacting surface of ubiquitin when complex with E2-25K and Ubc13 compared with the surface used for CR binding. (**b**,**c**) CR inhibits K48- (**b**) and K63-ubiquitinations (**c**). First, mono ubiquitin was mixed with UBA1, E2-25K or complex Mms2/Ubc13, and 0–500 µM CR in the presence or absence of ATP regeneration system. Then the mixture was incubated at 37 °C for 24 h. The reaction was stopped by adding 5X SDS loading buffer and the results were analyzed with 15% SDS-PAGE.

All of these compounds are symmetric, except for DB71. While CSB6B, EB, DB15, DB71, and TB each contain four sulfates, CR only has two. There are variations in the positions of the sulfonates and the other substituents among the compounds. CSB6B and EB showed similar binding affinities, as might be expected from their highly similar structures that differ only in the substituents on the central diphenyl moiety. The lower binding affinities of DB15 and TB suggest that the orientation of the sulfonates is important since their only difference from the higher affinity CSB6B and EB is the position and orientation of the sulfonates. More informative are the K_d_ and IC_50_ values of CR that only has one sulfonate on each naphthyl moiety, compared to the other compounds that have two. The comparable binding affinity of CR to those of CSB6B and EB suggests more clearly that the orientation of the sulfonates is crucial, rather than their number. In our previous study, owing to its four sulfonates and many other polar groups, CSB6B was suggested to act as the extracellular ubiquitin inhibitor with its low cell membrane permeability [35]. With the simplest structure, CR has the least of sulfonates and polar groups without affecting the inhibitory effect, and thus it was speculated that this compound might be able to penetrate the cell membrane. With this possibility, CR was chosen for further interaction study in comparison with CSB6B. From its similarity in modular structure to that of CSB6B, it is expected that the binding of CR is similar to that of CSB6B. Thus, to further analyze the interaction surface on ubiquitin with respect to CR and CSB6B, we carried out an NMR chemical shift mapping analysis of ubiquitin upon binding of the respective compounds (Figure 4 and Figures S2 and S3). Consistent with the previous result of the CSB6B-bound ubiquitin model constructed by docking study and molecular dynamic simulation [35], all the ubiquitin amino acid residues affected by CSB6B and CR are located surrounding the central canonical hydrophobic patch Ile44 on the ubiquitin surface. Furthermore, these amino acid residues were used as reference binding sites to construct a model of a CR-bound ubiquitin by docking and energy minimization, followed by binding stability verification with a molecular dynamic simulation (Figure S4). According to this model, it can be seen that CR has a similar mode of interaction with ubiquitin to that of CSB6B described previously [35]. The central canonical hydrophobic patch Ile44 on the ubiquitin surface has been shown to be the interacting interface for conjugating enzymes and deubiquitinases [5]. Therefore, this may explain the inhibitory activity of CSB6B and CR toward both tested DUBs and suggests that CSB6B and CR inhibit DUBs by binding to ubiquitin. The ubiquitin residues found to be affected by CR binding are also overlapped with the recognition sites for E2-25K, a K48-specific ubiquitin-conjugating enzyme, and Ubc13, a K63-specific ubiquitin-conjugating enzyme (Figure 5a). Therefore, CR is expected to show inhibitory activity toward these two enzymes, which was subsequently tested.

### 2.3. Congo Red Inhibits Ubiquitination In Vitro and, in the Cell

In vitro K48 and K63 polyubiquitination reactions were carried out using purified E2-25K or Ubc13 in the presence of increasing concentration of CR (Figure 5b,c). It was observed that both K48 and K63 polyubiquitination reactions were effectively inhibited by CR in a dose-dependent manner, demonstrating that upon binding to the interacting surface of ubiquitin used for ubiquitin processing enzymes, CR could effectively inhibit ubiquitin processing activity of those enzymes just as in the inhibition of DUB.

**Figure 6:**
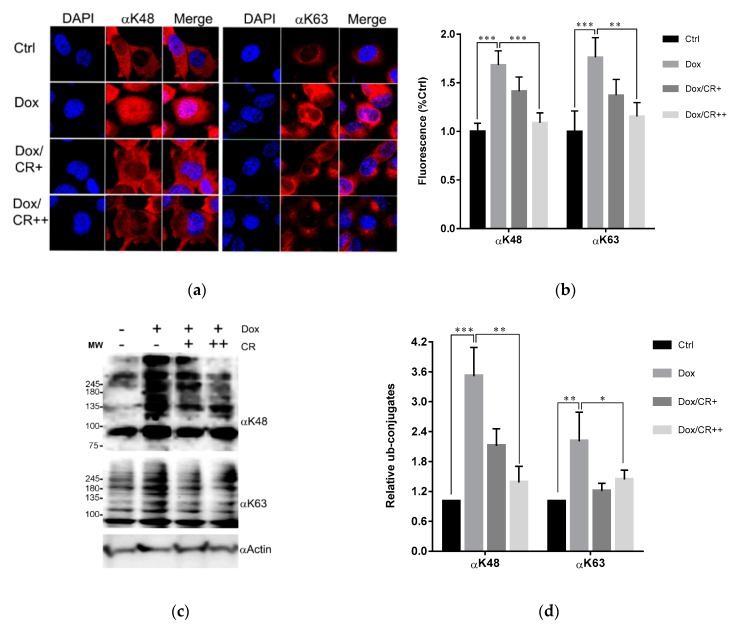
CR inhibits Dox-induced ubiquitination in the cells. (**a**) Immunofluorescence staining of H1299 cells treated with or without 5 (+) and 10 (++) μM CR for 12 h followed by addition of Dox to a final concentration of 5 μM, with incubation for 12 h. The cells were subjected to immunofluorescence staining using the antibodies against K48 (αK48) and K63 polyubiquitins (αK63). (**b**) K48 or K63 polyubiquitin level in Figure 6a was quantified by fluorescence signal. At least seven random regions acquired in confocal images were sampled and normalized against DAPI intensity for quantification. (**c**) Immunoblots of samples in Figure 6a using the indicated antibodies. (**d**) The band intensity of immunoblots in Figure 6c was quantified and displayed as the relative amount to the control. All error bars represent the standard deviation from at least four independent measurements. Statistical significance was calculated by the Student’s *t*-test (* *p* < 0.05, ** *p* < 0.01, *** *p* < 0.001).

With only two sulfonate groups and no hydroxyl or methoxy groups attached to the hydrophobic scaffold, as mentioned earlier, it was expected that CR might have a higher cell permeability than previously tested CSB6B [35]. Therefore, we tested the inhibitory activity of CR to the ubiquitination system intracellularly. Firstly, we tested the effect of CR on the ubiquitination state of normal cells. In vitro results indicated that CR could inhibit both ubiquitination and deubiquitination at the same time (Figure 3 and Figure 5). Therefore, immunoblots that show the increase or decrease of the polyubiquitin signals in the cells treated with CR would reflect the preferred direction being affected by CR. The effect of CR on the total ubiquitination of the normal cells resulted in a slight accumulation of the polyubiquitin chains (Figure S6), which suggests CR suppresses deubiquitination more than it does ubiquitination under this condition. However, this suppression does not affect the cell’s viability (Figure S7). Next, we tested the effect of CR when one of the two opposite pathways was activated. First, CR was tested on the activated ubiquitination in cells. It has been reported that the ubiquitin system is activated when cancer cells are treated with Dox [25]. Hence, we chose doxorubicin (Dox)-treated cancer cells as the model for this study. Thus, we incubated the CR-pretreated and non-pretreated H1299 cells with 5 µM Dox and determined the levels of ubiquitination in the cells by immunofluorescence staining, using antibodies specific to K48- and K63-polyubiquitin. Consistent with previous reports, non-pretreated cells showed enhanced K48- and K63-polyubiquitination upon the treatment with Dox, suggesting that ubiquitin system was activated. However, such enhanced ubiquitination was not observed in CR-pretreated cells (Figure 6a,b), implying that CR suppressed Dox-triggered ubiquitination in both K48 and K63. These in-cell results were consistent with the results of Western blot analysis (Figure 6c,d). Second, CR was tested on the supplemented deubiquitination activity of cells. The activity of USP4 on its substrate β-catenin [38] was selected for this study (Figure S5). A top-flash activity assay of ß-catenin in the absence or presence of USP4 with different concentrations (0–30 μM) of CR was conducted to visualize the effect of USP4 and CR to the transcriptional activity of β-catenin. Consistent with a previous study [38], β-catenin transcriptional activity was enhanced upon co-transfection with USP4. Expectedly, this enhancement was antagonized by CR treatment (Figure S5a). Western blot analysis with indicated antibodies has also confirmed the antagonistic effect of CR to the USP4 dependent stabilization of β-catenin at the protein level (Figure S5b). Taken together, these results suggest that under these experimental conditions, CR can inhibit either ubiquitination or deubiquitination depending on which pathway is abnormally activated and more pronounced.

### 2.4. Congo Red Enhances Doxorubicin Sensitivity of the Cancer Cell

**Figure 7:**
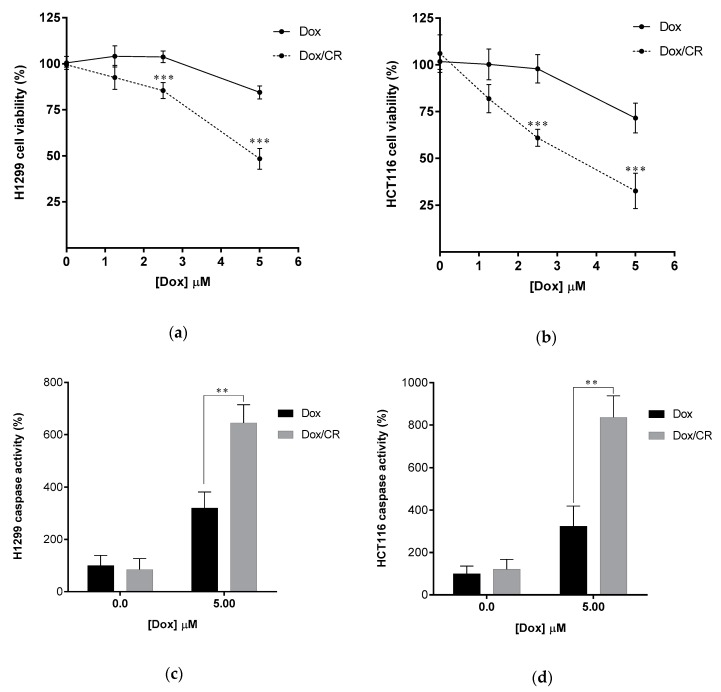
CR enhances Dox sensitivity of cancer cells. (**a**,**b**) Enhancement of Dox-induced apoptosis of cancer cells by CR treatment. Dox–induced apoptosis of H1299 (**a**) and HCT116 (**b**) was quantified by colorimetric cell viability after treating 10 µM CR for 12 h followed by 12h treatment of Dox (0.0–5.0 µM). (**c**,**d**) Enhanced caspase activity of Dox-treated H1299 (**c**) and HCT116 (**d**) cells by 10 µM CR. Caspase activity was quantified by luminogenic caspase activity assay. Data are displayed as the percentage of control samples (0.0 µM Dox). All error bars represent the standard deviation from at least four independent experiments. Statistical significance was calculated by the Student’s *t*-test (** *p* < 0.01, *** *p* < 0.001).

There is growing evidence that the levels of ubiquitin and ubiquitin chains are elevated in several cancers [39,40,41]. Therefore, the downregulation of ubiquitin levels or inhibition of Ub-chain formation could lead to positive outcomes in cancer treatment. Consistent with this, the downregulation of ubiquitin by UBB knockdown has been found to have an anticancer effect in several cancer cell lines and xenograft mice [42]. Moreover, the knockdown of two ubiquitin-encoding genes including polyubiquitin B (UBB) and polyubiquitin C (UBC) enhanced the efficacy of therapeutic irradiation against cancer [43]. In addition, it has been reported that the activated ubiquitin system in Dox-treated cancer cells promoted the degradation of the inhibitor for NF-κB, IκBα, possibly by K48-polyubiquitination [44,45] leading to the reduced anticancer effect of Dox. Indeed, proteasome inhibitors enhanced Dox sensitivity in several cancer cells [46,47,48]. Moreover, K63 polyubiquitination has been implicated to be responsible for Dox-mediated NF-κB activation and DNA damage repair pathways [3,27,29] and suggested to be the target for suppression to gain enhancement of Dox sensitivity [44]. These findings imply that suppression of either K48 or K63 polyubiquitination might enhance Dox therapy. To test this possibility, we studied the effect of CR and NSC697923, a specific inhibitor of Ubc13, a K63-specific ubiquitin-conjugating enzyme, [49,50] which plays a major role in both NF-κB activation and DNA damage repair pathway [51,52,53] with Dox. The effect of CR and NSC697923 on Dox-induced apoptotic cell death was investigated. CR or NSC697923 was used to treat H1299 cells and HCT116 cells (colon cancer cell line) at the none-toxicity concentration (10 µM for CR and 5 µM for NSC697923), determined by cytotoxicity assays (Figure S7), in combination with different concentrations of Dox. Subsequently, cell viability (Figure 7a,b and Figure S8a,b) and the activity of caspases (Figure 7c,d and Figure S8c,d) were measured to determine the progress of apoptosis. The amount of Dox-induced cell death was proportional to the Dox concentration (1.25 to 5.0 μM) (Figure 7a,b and Figure S8a,b). However, CR and NSC967923 significantly enhanced Dox-induced cell death in both cell lines (Figure 7a,b and Figure S8a,b), suggesting that the two compounds acted similarly suppressing ubiquitination, particularly, Ubc13 mediated K63-ubiquitination, can be used as a novel method to enhance Dox therapy. However, we noted that NSC697923 had only a synergistic effect at low concentrations of Dox (Figure S8b) in colon cancer cells, HCT116. Taken together, these results strongly suggest that the efficacy of Dox chemotherapy can be enhanced by the intracellular action of CR, which disrupts the recognition of ubiquitin.

## 3. Discussion

The interacting surfaces of ubiquitin are commonly located surrounding the three central hydrophobic patches including Ile44 (Leu8, Ile44, His68 and Val70), Ile36 (Ile36, Leu71, and Leu73), and Phe4 (Gln2, Phe4 and Thr14), and TEK-box (Lys6, Lys11, Thr12, Thr14, and Glu34) [5]. Among them, Ile44 is important for conjugation and deconjugation since most of ubiquitin processing enzymes including E2s and DUBs interact with this canonical hydrophobic patch [54]. Ile36 is required for interaction between monomer units in the polyubiquitin chain and for recognition by HECT domain of E3s, and some DUBs [5,54], whereas the Phe4 patch is indispensable for the mono ubiquitin-mediated protein trafficking and endocytosis [54], and TEK-box plays a role in mitotic degradation [5]. In this study, we found that the scaffold of selected sulfonated aryl diazo compounds inhibits DUBs by direct binding to ubiquitin instead of binding to the enzyme. In addition, we tested the binding affinities of some of the structurally related sulfonated diazo compounds for ubiquitin along with DUB inhibitory activity using USP5 and UCHL1, and found that the position and orientation of sulfonates, rather than their number, play an important role in binding and inhibitory activities. We also found that the compounds have stronger inhibitory activity against USP5 than that to UCHL1. The difference between these two enzymes is the ubiquitin-interacting surface. Despite the similar binding to C-terminal motif and the Ile36 patch of ubiquitin, USP5 mainly contacts with the Ile44 patch, whereas UCHL1 does not (Figure S9). Therefore, it is reasonable to predict that these chemicals will inhibit DUBs by blocking the hydrophobic surface surrounding Ile44 and the C-terminal motif of ubiquitin. Furthermore, Congo red (CR), the simplest and reasonably active compound, was chosen for an NMR mapping assay and the results confirmed the above prediction. This result also indicated that CR could also inhibit the ubiquitination enzymes in addition to DUBs. Accordingly, CR efficiently inhibited K48 and K63 polyubiquitination carried out by E2-25K and Ubc13 respectively (Figure 5).

In our previous study, owing to its many hydrophilic and anionic groups, CSB6B was not able to penetrate the cell membrane and therefore could only work as an extracellular inhibitor [35] Taking advantage of such characteristics of CSB6B, it was used to control the extracellular function of ubiquitin. In contrast, CR showed a better cell permeability, and thus it could act as an intracellular agent affecting the ubiquitination (Figure 6). It has been reported that Dox-treated cancer cells develop resistance to Dox by activating the ubiquitin-proteasome system [44,45] leading to the activation of NF-κB and DNA repair pathway, and thus Dox resistance can be antagonized by proteasome inhibitors [46,48]. Using this strategy, we demonstrated that CR could increase sensitivity of cancer cells to Dox by suppressing both K48- and K63-ubiquitination activated by Dox treatment. This was inconsistent with the effect of NSC697923, an approved inhibitor of Ubc13, the key K63-specific conjugating enzyme, which mediates activation of both NF-κB and DNA damage repair pathways. Both compounds showed a similar synergistic anti-cancer effect with Dox (Figure 7 and Figure S8). Taken together, these results suggest that binding to the interacting surface of ubiquitin could disrupt the protein–protein interaction involving ubiquitin, leading to the same effect as the inhibition of ubiquitin processing enzymes, whether conjugating or hydrolyzing. Moreover, through these examples, we clearly demonstrated for the first time that small molecules binding to ubiquitin could display therapeutic effects, and the molecular scaffold of CSB6B and CR may provide the extracellular or intracellular specific inhibition of the ubiquitin pathway.

## 4. Materials and Methods

### 4.1. Cell Lines, Chemicals, Antibodies, and Plasmids

HEK293T, H1299, and HCT116 cell lines were purchased from ATCC. The cells were handled according to the supplier’s instructions. All chemicals used in this study were purchased from Sigma-Aldrich, unless otherwise specified. EZ–Cytox cell viability assay kit was purchased from Daeil Lab Service (Chungbuk, South Korea). Caspase Glo^TM^ 3/7 assays kit was purchased from PROMEGA. Antibodies against ubiquitin (cat # sc-271289), and β-actin (cat # sc-47778) were purchased from Santa Cruz, K48- and K63 specific ubiquitin antibodies (cat # 05-1307 and 05-1308) were purchased from Merck Millipore.

### 4.2. Protein Preparation

All the proteins were prepared as described previously [35]. ^15^N-labeled ubiquitin was prepared from *E. coli* cells cultured in M9-based minimal medium supplemented with ^15^NH_4_Cl (Cambridge Isotope Laboratories Inc, Massachusetts, MA, USA). The sample for NMR experiment was dialyzed into NMR buffer containing 25 mM HEPES (pH 7.0), 150 mM NaCl, and 1 mM EDTA.

### 4.3. Enzyme Activity Assay

For in vitro ubiquitination assays, 500 µM of ubiquitin was used for each ubiquitination reaction as described previously [35], in the absence or presence of 50–500 µM of CR. The reactions were incubated at 37 °C for 4 h and 16 h for K63- and K48 polyubiquitination respectively. The reaction was terminated by adding 5X SDS sampling buffer and results were subjected to 15% SDS-PAGE followed by Coomassie Blue staining for analyses. For deubiquitination assays, 0.6 µM of USP5 or 8 µM of UCHL1 and 5 µM of GST-Ubiquitin-HA were used for each deubiquitination reaction in the presence of 0–45 µM of indicated compound in the buffer condition as described previously [35]. The generation of GST-ubiquitin from GST-Ubiquitin-HA as the mobility shifts were analyzed on 12.5% SDS-PAGE visualized by Coomassie blue staining. Band intensities were quantified by ImageJ [55] and used to generate the IC_50_ curves (log inhibitor vs. normalized response with variable slope) by GraphPad Prism version 6.0 for Windows (GraphPad Software, San Diego, CA, USA, www.graphpad.com).

### 4.4. Native Polyacrylamide Electrophoresis for Checking Interaction of Ubiquitin and Chemicals

Purified ubiquitin was mixed with chemicals with indicated molar ratio as mentioned in the descriptions of the specific experiments in distilled water and incubated for 1 h on ice before loading onto 10% native Tris-glycine polyacrylamide gels for visualization of the interaction.

### 4.5. Immunofluorescence Staining and Image Analysis

Cells were seeded in the SPL 4 wells cell culture chamber slide 24 h before experiment. Cells were treated with an indicated compound for an indicated time. The cells were fixed with 4% formaldehyde for 15 min followed by three times of PBS washing. Cells were permeabilized by incubation with PBS containing 0.5% Triton-X100 and 5% FBS for 30 min, and then blocked with blocking buffer (PBS, 5% FBS, 0.3% Triton X-100) for 1.5 h. Subsequently, cells were incubated with primary antibody at 1/200 dilution in blocking buffer overnight and then washed with blocking buffer five times (5 min each) before incubation with fluorescence-conjugated secondary antibody at 1/200 dilution in blocking buffer for 1 h. Cells were then washed with blocking buffer five times (5 min each) before incubation with DAPI for 10 min and three times washing with PBS. Slides were covered with the coverslip in the presence of mounting solution (cat # 475904, MOWIOL) and subjected to confocal imaging. Images acquired in confocal imaging were subjected to fluorescence intensity analysis using ImageJ [55]. Fluorescence signal representing a particular ubiquitin chain level was analyzed and normalized with the intensity of DAPI. Data were analyzed using GraphPad Prism version 6.0 for Windows with two tail t-test for statistical data analysis.

### 4.6. Cell Viability and Caspase Assays

Cell viability assays were performed as described previously [35]. For the caspase assays, the media were removed, and cells were lysed in 200 µL lysis buffer containing 50 µL Caspase Glo 3/7 solution and incubated for 15 min. Results were observed by measuring the luminescence signal using KOMM-Nr (Berthold Technologies, Bad Wildbad, Germany) and normalized with the total protein concentration measured by the Bradford assay. Data were analyzed using GraphPad Prism version 6.0 for Windows with two tail t-test for statistical data analysis

### 4.7. Sample Preparation for NMR Experiments

^15^N-labeled ubiquitin was prepared from *E. coli* cells cultured in M9-based minimal medium supplemented with ^15^NH_4_Cl (Cambridge Isotope Laboratories Inc). The procedure for purifying NMR samples is the same as that for the preparation of non-labeled samples. The sample for NMR experiment was dialyzed into NMR buffer containing 25 mM HEPES (pH 7.0), 150 mM NaCl, and 1 mM EDTA.

### 4.8. NMR Experiment

All ^1^H,^15^N-HSQC spectra were obtained at 25 °C using a gNhsqc pulse sequence on a Varian Unity Inova 500 MHz spectrometer at the National Institutes of Environment and Health Science (National Institutes of Health in North Carolina, Durham, NC, USA). The chemical shifts of amide backbone were assigned by the simulation and extraction of the ubiquitin chemical shift data deposited at the Biological Magnetic Resonance Data Bank (BMRB entry number: 6457). Chemical shift perturbation (CSP) experiments using ^1^H,^15^N-HSQC spectra were performed in NMR buffer containing 10% D_2_O. The ^1^H,^15^N-HSQC spectra of 100 μM ^15^N-labeled ubiquitin were monitored by titrating Congo red, respectively, with chemical/protein ratios of 0.2, 0.5, 1, and 2. The intensity reduction ratio (ΔI) of peaks observed in the intermediate exchange rate between free and bound forms, in the NMR time scale, was employed to determine the binding surface of ubiquitin to each chemical. It was calculated by the following equation:

ΔI = I/I_0_ (1)

(I: intensity of bound-form, I_0_: intensity of free-form)

For the characterization, ΔI was normalized to the normalized intensity reduction ratio (I_N_). It was obtained by the following equation:

I_N_ = (ΔI_obs_ − ΔI_min_)/(ΔI_max_ − ΔI_min_) (2)

(ΔI_obs_: observed ΔI, ΔI_max_: highest ΔI, ΔI_min_: lowest ΔI)

Processing and analysis of the NMR spectra were performed by NMRPipe [56] and Sparky 3.115 [57], respectively.

### 4.9. Luciferase Reporter Assays

Luciferase reporter assays were performed as described previously [38]. Briefly, HEK293T cells were seeded in 12-well plates at 1 × 10^5^ cells per well and incubated for 24 h before being transfected with either SRT-USP4 or Myc-β-catenin expression plasmids, along with the T cell factor (TCF) reporter plasmid (TOP flash) driving the expression of the luciferase gene. Equal amounts of total DNA in transfections were maintained by adding the control plasmids in each transfection experiment. 24 h post-transfection, cells were treated with CR (0–30 µM) for 24 h. After that, cells were harvested for luciferase reporter assays. Luciferase activity was measured according to the manufacturer’s instructions (Promega, Madison, WI, USA).

### 4.10. Molecular Docking and Molecular Dynamic Simulation

Structure of ubiquitin monomer was obtained from the PDB (ID: 2Y5B) and SDF files of Congo red (CR) were obtained from NCBI PubChem database (PubChem CID: 11314) [https://pubchem.ncbi.nlm.nih.gov/]. Preparing of both protein and ligand structures and the molecular docking and molecular dynamic simulation were performed as described previously [35].

### 4.11. Intrinsic Fluorescence Quenching Assays

Intrinsic fluorescence quenching assays were performed as described previously. [35] Briefly, intrinsic fluorescence quenching of ubiquitin by indicated compounds was observed by measuring the excitation and emission wavelength at 274 nm and 305 nm respectively of the mixture of 50 µM purified ubiquitin and 0–500 µM compound. Data were fitted to nonlinear regression curves with the equation described previously [35].

F = −∆ε·[PC] + F_o_ (3)

b = (P_o_ + C_o_ + K_d_) (4)

c = P_o_·C_o_ (5)

[PC] = [b − (b^2^ − 4·c)^1/2^]/2 (6)

where F_o_ and F are the normalized fluorescence intensity of the mixture of ubiquitin without the compound and with different concentrations of the compound, respectively. ∆ε is a difference in the extinction coefficient of the compound between their free and complex forms. [PC] is the concentration of protein/compound complex. P_o_ and C_o_ are the added concentration of ubiquitin and the compound, respectively in each experiment. K_d_ is the dissociation constant.

### 4.12. Statistical Analysis

Results were normalized by control value and represented as the mean ± standard error of the mean. All experiments were repeated at least three times unless otherwise mentioned. Statistical comparisons between groups were determined by Student’s t-test. If p-values are less than 0.05, they are considered to be statistically significant and indicated in the graph. The statistical significance of the normalized values was indicated by “*” (*p* < 0.05), “**” (*p* < 0.01), and “***” (*p* < 0.001).

## Figures and Tables

**Table 1:** Dissociation constant (K_D_) upon binding to ubiquitin of different compounds.

Compounds	K_d_ (µM)
CSB6B	12.0 ± 0.6781
EB	13.0 ± 0.6887
CR	14.4 ± 1.2739
DB71	20.0 ± 0.8352
DB15	60.3 ± 2.3011
TB	78.1 ± 4.0244

**Table 2:** Half maximal inhibitory concentration (IC_50_) of different compounds toward DUB activity of USP5 and UCHL1.

Compounds	IC50 (µM) USP5	IC50 (µM) UCHL1
CSB6B	6.52 ± 0.786	11.2 ± 1.37
EB	9.33 ± 1.577	18.1 ± 2.09
CR	13.4 ± 2.640	26.9 ± 2.99
DB71	13.72 ± 2.740	33.7 ± 4.05
DB15	32.52 ± 5.00	89 ± 15.6
TB	57.71 ± 13.13	100.2 ± 19.1

## Supplementary Materials

The following are available online at. Figure S1: Activities of USP5 (a) and UCHL1 (b) upon treatment with different ubiquitin-binding compounds; Figure S2: Intensity reduction of ubiquitin residues upon the titration of Congo Red; Figure S3: The interacting surface of ubiquitin used for binding with CSB6B; Figure S4: The interaction mode of CR-bound ubiquitin; Figure S5: CR inhibits USP4 deubiquitinase activity on its substrate, ß-catenin on the TOP flash reporter activity; Figure S6: Effect of CR on the ubiquitination state of none-treated cells; Figure S7: Cytotoxicity of CR to cancer cells; Figure S8: NSC697923 enhances Dox sensitivity of cancer cells; Figure S9: Different interacting interface of ubiquitin used for forming complex with USP5 (left) and UCHL1 (right).
