# Supplementary material for: Suppression of the Ubiquitin Pathway by Small Molecule Binding to Ubiquitin Enhances Doxorubicin Sensitivity of the Cancer Cells
Source: Molecules. 2019 Mar 19;24(6):1073. doi: 10.3390/molecules24061073 (PMC6471062; doi:10.3390/molecules24061073)
Supplement: Supplementary file 1 [file molecules-24-01073-s001.pdf]

Supplementary Information

Article

Suppress the ubiquitin pathway by small molecule binding to ubiquitin enhances doxorubicin sensitivity of the cancer cells

Thanh Nguyen [1], Minh Ho [1], Kyungmin Kim [2], Sun-Il Yun [1], Pushpak Mizar [3], James W Easton [3], Seung Seo Lee [3,*] and Kyeong Kyu Kim [1,*]

[1] Department of Molecular Cell Biology, Sungkyunkwan University School of Medicine, Suwon 440-746, Korea; ntth@skku.edu (T.N.); honguyenanhminh@gmail.com (M.H.); sipoppy@hanmail.net (S.I.Y.)

[2] Genome Integrity and Structural Biology Laboratory, NIEHS, National Institutes of Health, Research Triangle Park, NC 27709, USA; sbl.kmkim@gmail.com (K.K.)

[3] Chemistry, Faculty of Engineering & Physical Sciences, University of Southampton, Highfield, Southampton, SO17 1BJ, UK; P.Mizar@soton.ac.uk (P.M.); jwe1g13@soton.ac.uk (J.W.E.)

* Correspondence: S.S.Lee@soton.ac.uk (S.S.L.); kyeongkyu@skku.edu (K.K.K.); Tel.: +44-2380-592987 (S.S.L.); +82-31-299-6136 (K.K.K.)

Supplemental Figures

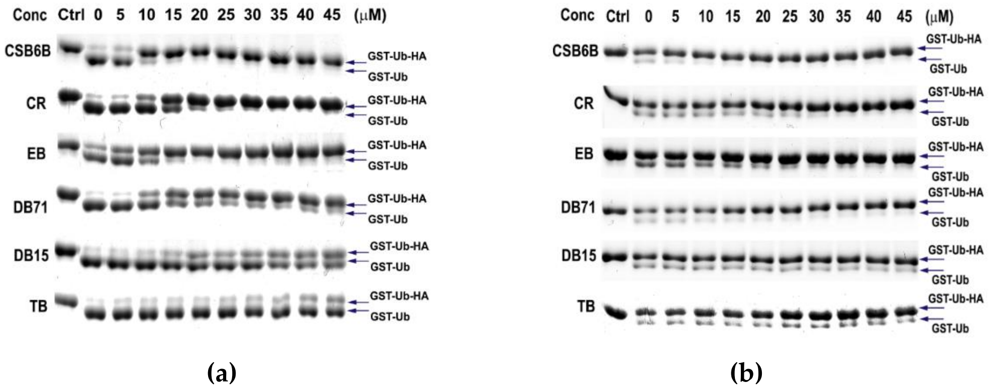

Figure S1. Activities of USP5 (a) and UCHL1 (b) upon treatment with different ubiquitin-binding compounds. Deubiquitination activity of USP5 and UCHL1 were measured by monitoring the cleavage of HA from GST-Ub-HA resulted in mobility shift of the product bands (GST-Ub) compare to no enzyme treatment (Ctrl) in SDS-PAGE. Inhibitory activity of different ubiquitin binding-compounds were visualized by the reduction of product bands upon treated with an increasing concentration of compounds (0-45 μM).

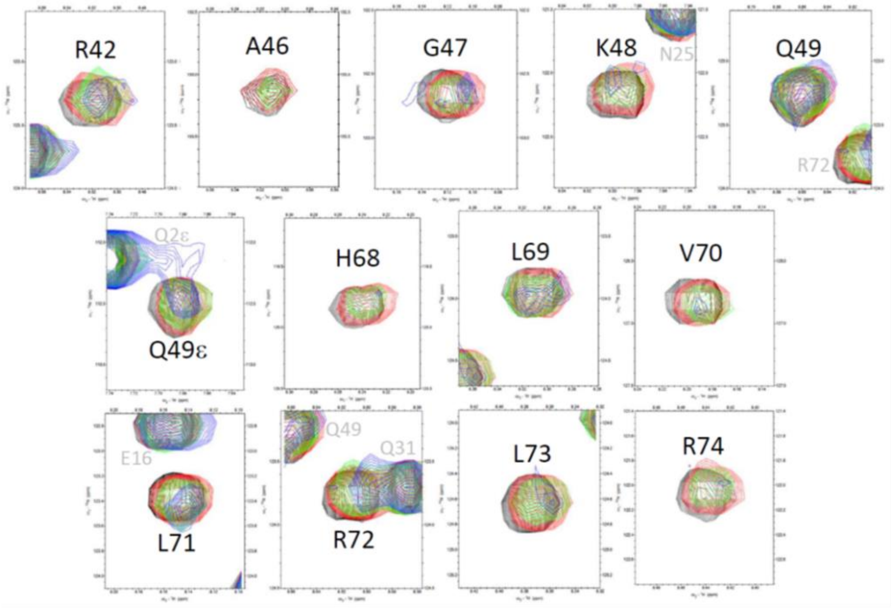

Figure S2. Intensity reduction of ubiquitin residues upon the titration of Congo Red. The $^{1}H,^{15}N$-HSQC spectra of 13 residues [12 from the amide backbone (R42, A46, G47, K48, Q49, H68, L69, V70, L71, R72, L73, and R74) and one from a side chain (Q49ε)] exhibiting severe intensity reduction upon Congo Red ($I_N < 0.25$) titration (black: 100 μM ubiquitin alone; red: with 20 μM ligands; green: with 50 μM ligands; blue: with 100 μM ligands).

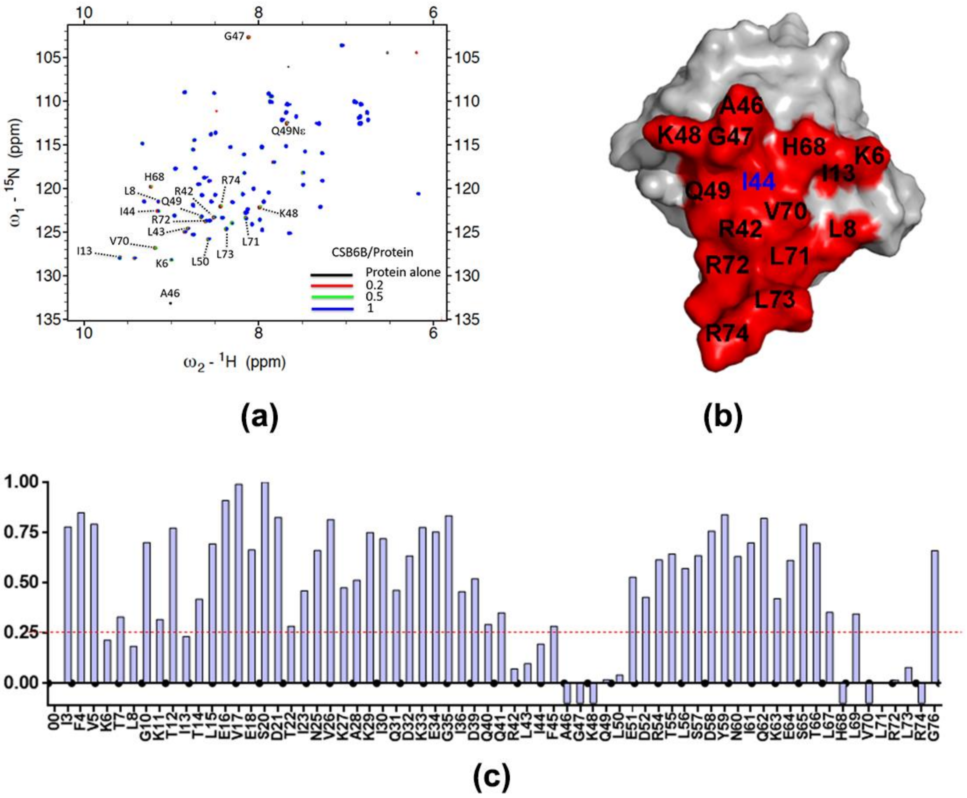

Figure S3. The interacting surface of ubiquitin used for binding with CSB6B. (a) ^{1}H,^{15}N-HSQC spectra of ^{15}N-labeled ubiquitin upon treatment of CSB6B at different concentration: Black, 100 μM ^{15}N-ubiquitin alone; Red, with 20 μM CSB6B; Green, with 50 μM CSB6B; Blue, with 100 μM CSB6B. The peaks with chemical shift or line broadening are indicated by dotted lines and labeled. **(b)** The ubiquitin interacting surface used for CSB6B binding. The residues disturbed upon treatment with CSB6B were indicated in red color with labeled on the surface-filling model of Ub. The center of the canonical hydrophobic patched, Ile44 is labeled in blue. **(c)** Normalized intensity reduction ratio (I_N) of each residue upon treatment of CSB6B at 1:1 ratio. The value below zero (-0.1) indicates the disappearing residues.

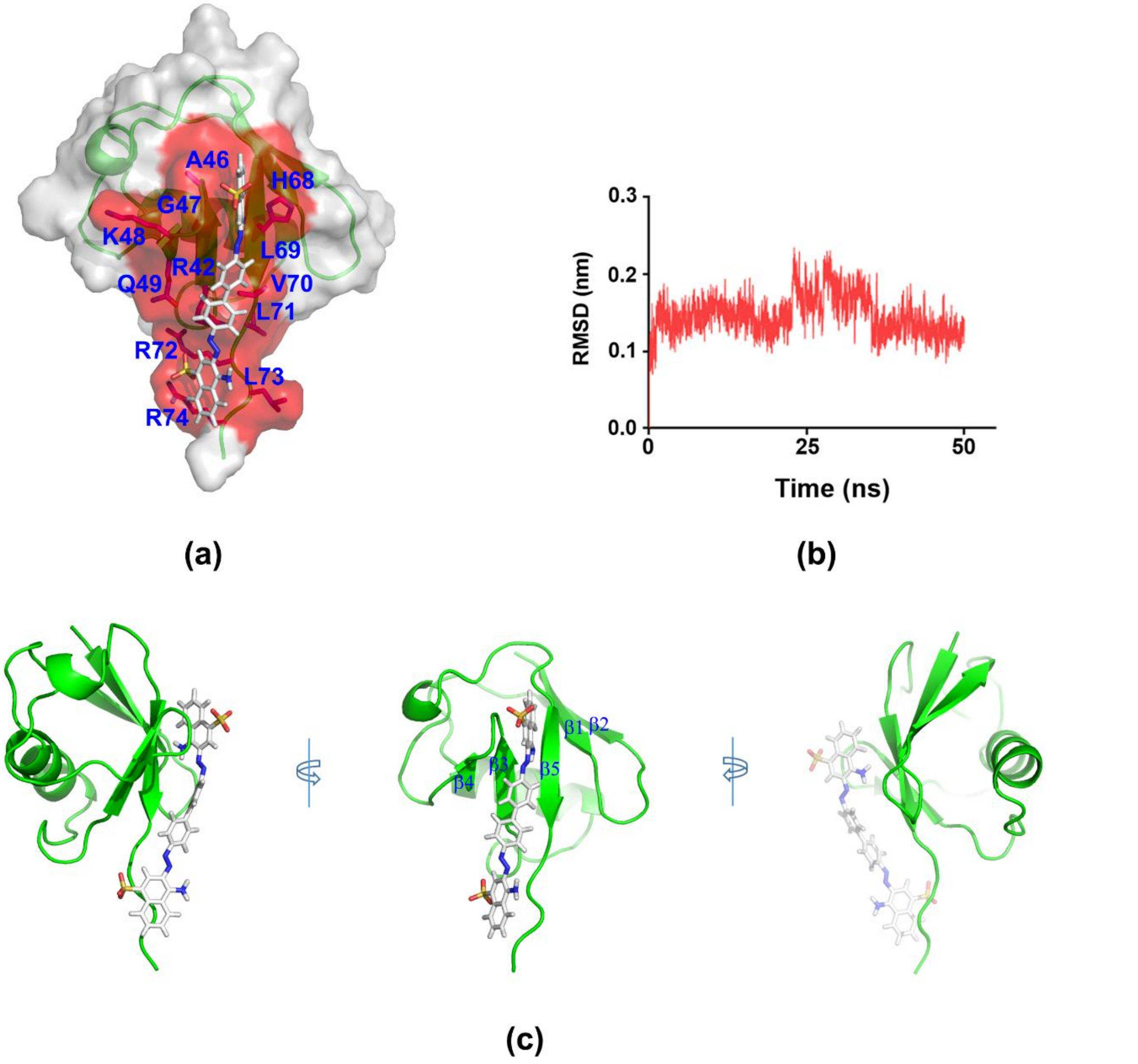

Figure S4. The interaction mode of CR-bound ubiquitin. (**a**) Model of CR-bound ubiquitin complex constructed by docking. CR is drawn as white stick model on the surface – filling model of ubiquitin with the interacting surface labeled in red. The residues found in NMR experiment were drawn in pink sticks with indicated labeling. (**b**) The RMSD over simulation time of CR in complex with ubiquitin was presented. Fluctuation less than 0.1 nm in RMSD over simulation time (50 ns) confirming the stability of the complex model. (**c**) Different side views of the Ub/CR complex model.

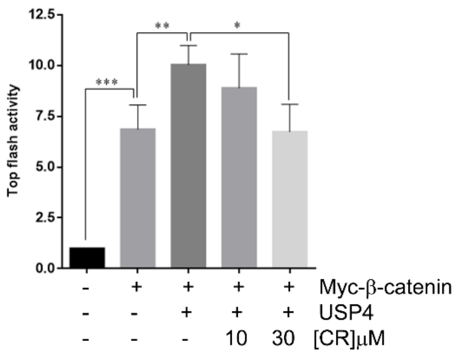

(a)

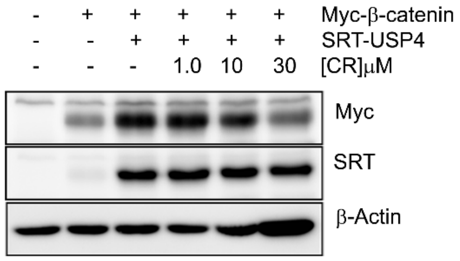

(b)

Figure S5. **CR inhibits USP4 deubiquitinase activity on its substrate, ß-catenin on the TOP flash reporter activity. (a)** Cells were co-transfected with either SRT-USP4 or Myc-β-catenin expression plasmids, and TOP flash reporter plasmid containing a β-catenin-induced TCF reporter. 24-h post-transfection, CR was added at increasing concentrations (0 – 30 μM) and the cells were further cultured for 24 h. A top-flash activity assay of ß-catenin was conducted and (**b**) Western blot analysis with the indicated antibody was performed in parallel. All error bars represent the standard deviation from at least 3 independent experiments. *, P<0.05; **, P<0.01; ***, P<0.001.

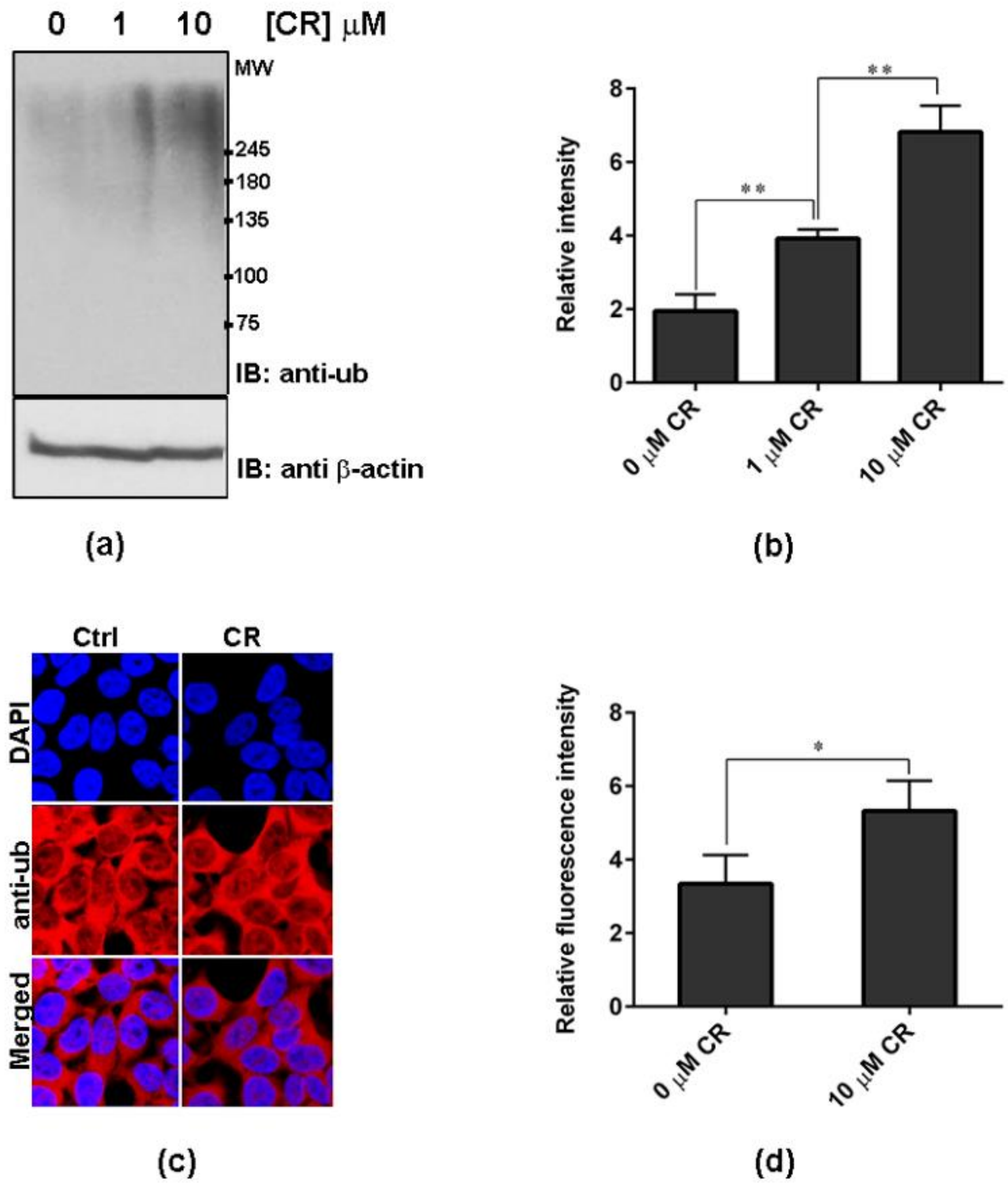

Figure S6. Effect of CR on the ubiquitination state of none-treated cells. (**a**) H1299 cells treated with 1.0 and 10 μM CR for 12 h. The cells were subjected to immunoblots using the anti-ubiquitin antibody (P4D1, Santa-Cruz, cat #8017) or anti-β-actin (Santa-Cruz, cat #sc-47778). (**b**) The intensity of immunoblots in (**a)** was quantified and displayed as the relative amount to the control (actin) (**c**) Immunofluorescence staining of H1299 cells treated as in (**a**). However, concentration of CR was fixed at 10 μM. (d) The intensity of immunofluorescences staining in (**c)** were quantified and displayed as the relative amount to the control (DAPI). All error bars represent the standard deviation from at least 3 independent measurements. *, P<0.05; **, P<0.01; ***, P<0.001.

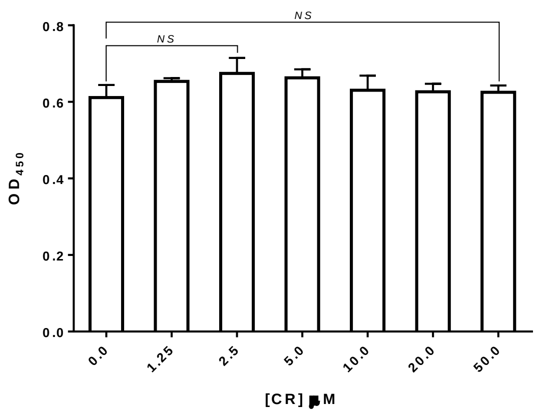

Figure S7. Cytotoxicity of CR to cancer cells. H1299 cells were cultured in 96-well plates. CR was added to final concentrations of 0.0 – 50.0 μM, and the cells were incubated for 24 h followed by cell viability assay. All error bars represent the standard deviation from at least 4 independent experiments. Statistical comparisons between groups were determined by Student's t-test. If p-values are less than 0.05, they are statistically significant. *NS*, not significant.

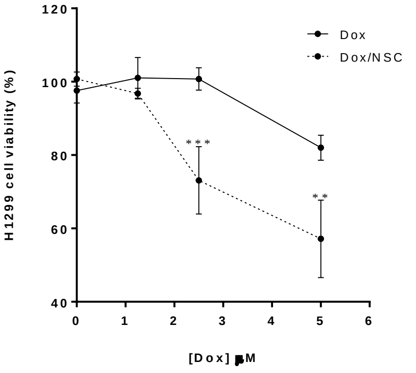

(a)

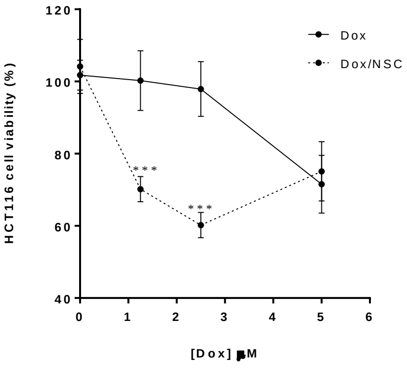

(b)

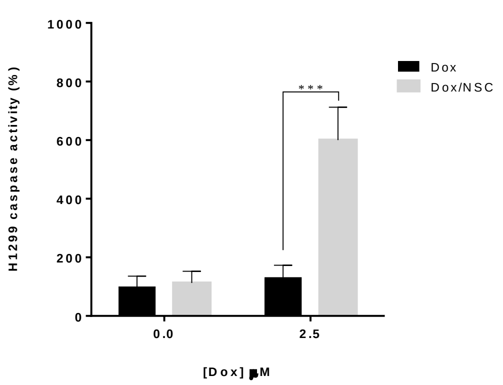

(c)

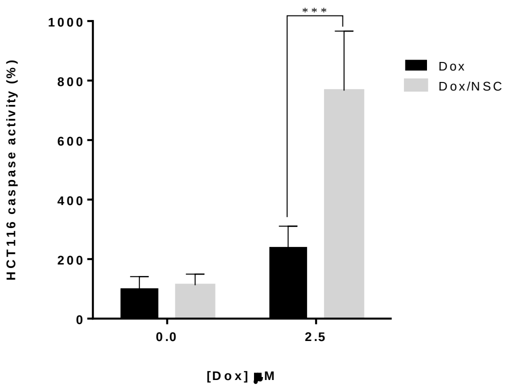

(d)

Figure S8. **NSC697923 enhances Dox sensitivity of cancer cells. (a, b)** Effect of NSC697923 to cancer cells' viability in Dox treatment. Dox–induced cell death of H1299 (**a**) and HCT116 (**b**) was quantified by colorimetric cell viability assay after treating 5 µM NSC697923 for 12 h followed by 12h treatment of Dox (0.0 – 5 µM). (**c, d**) Enhanced caspase activity of (0-2.5 µM) Dox-treated H1299 (**c**) and HCT116 (**d**) cells by 5 µM NSC697923. Caspase activity was quantified by luminogenic caspase activity assay. All error bars represent the standard deviation from at least 4 independent experiments. *, P<0.05; **, P<0.01; ***, P<0.001.

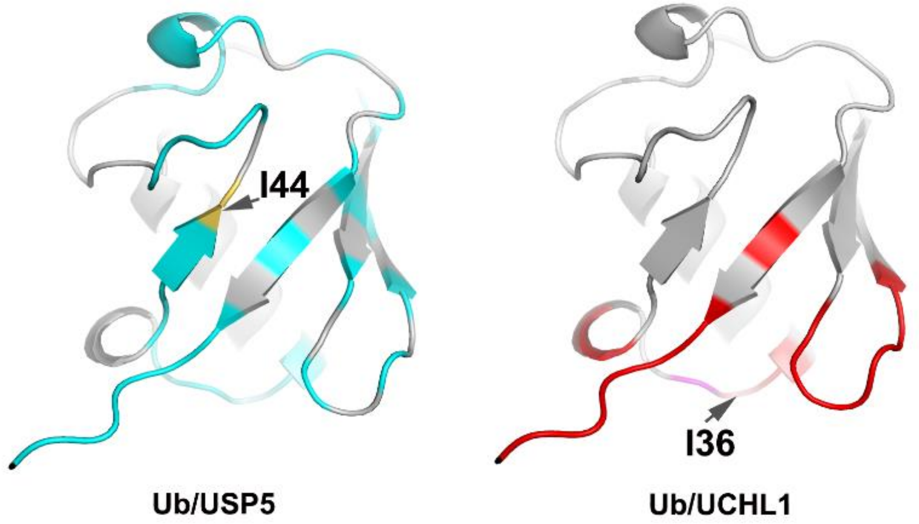

Figure S9. Different interacting interface of ubiquitin used for forming complex with USP5 (left) and UCHL1 (right). Complex structure of Ub/USP5 (PDB #3IHP) and Ub/UCHL1 (PDB #3IFW) were subjected to protein-protein interaction analysis using PDBsum (http://www.ebi.ac.uk/). Information of interacting residues of ubiquitin in each complex was used to label with indicated color on the ubiquitin structure; cyan, Ub/USP5; red, Ub/UCHL1. Ile 44 and Ile 36 are indicated with dark arrow.
